# Different Effects of Guanine Nucleotides (GDP and GTP) on Protein-Mediated Mitochondrial Proton Leak

**DOI:** 10.1371/journal.pone.0098969

**Published:** 2014-06-06

**Authors:** Andrzej M. Woyda-Ploszczyca, Wieslawa Jarmuszkiewicz

**Affiliations:** Department of Bioenergetics, Adam Mickiewicz University, Poznan, Poland; Instituto Nacional de Cardiologia, Mexico

## Abstract

In this study, we compared the influence of GDP and GTP on isolated mitochondria respiring under conditions favoring oxidative phosphorylation (OXPHOS) and under conditions excluding this process, i.e., in the presence of carboxyatractyloside, an adenine nucleotide translocase inhibitor, and/or oligomycin, an F_O_F_1_-ATP synthase inhibitor. Using mitochondria isolated from rat kidney and human endothelial cells, we found that the action of GDP and GTP can differ diametrically depending on the conditions. Namely, under conditions favoring OXPHOS, both in the absence and presence of linoleic acid, an activator of uncoupling proteins (UCPs), the addition of 1 mM GDP resulted in the state 4 (non-phosphorylating respiration)-state 3 (phosphorylating respiration) transition, which is characteristic of ADP oxidative phosphorylation. In contrast, the addition of 1 mM GTP resulted in a decrease in the respiratory rate and an increase in the membrane potential, which is characteristic of UCP inhibition. The stimulatory effect of GDP, but not GTP, was also observed in inside-out submitochondrial particles prepared from rat kidney mitochondria. However, the effects of GDP and GTP were more similar in the presence of OXPHOS inhibitors. The importance of these observations in connection with the action of UCPs, adenine nucleotide translocase (or other carboxyatractyloside-sensitive carriers), carboxyatractyloside- and purine nucleotide-insensitive carriers, as well as nucleoside-diphosphate kinase (NDPK) are considered. Because the measurements favoring oxidative phosphorylation better reflect *in vivo* conditions, our study strongly supports the idea that GDP cannot be considered a significant physiological inhibitor of UCP. Moreover, it appears that, under native conditions, GTP functions as a more efficient UCP inhibitor than GDP and ATP.

## Introduction

The mitochondrial proton electrochemical gradient generated by the respiratory chain pumps drives ATP synthesis as a result of oxidative phosphorylation (OXPHOS). However, F_O_F_1_-ATP synthase is not the only factor consuming this gradient. The inner mitochondrial membrane (IMM) possesses many protein carriers included in the mitochondrial anion carrier protein family, among which uncoupling proteins (UCPs) and adenine nucleotide translocase (ANT) both have an affinity to bind purine nucleotides as well as to mediate non-phosphorylating proton leak [1], [2]. UCPs have been identified across eukaryotes, including eukaryotic microorganisms, plants, and vertebrate as well as invertebrate species [3]. UCPs specialize in proton translocation from the intermembrane space into the mitochondrial matrix, a process that is not related to ATP synthesis and dissipates energy as heat. The abundant expression and action of UCP1, the first described UCP, in brown adipose tissue of small, hibernating, and cold-acclimated mammals are responsible for the thermogenic properties of this tissue. However, the role of UCP isoforms in unicellular organisms, such as the amoeba *Acanthamoeba castellanii*, and in the non-thermogenic cells of plants and animals remains unclear [3]. As UCPs, similar to artificial uncouplers, decrease the mitochondrial membrane potential (ΔΨ) and thus the redox state of respiratory chain electron carriers, their action should lower the production of reactive oxygen species [4]. Although many data indicate the antioxidative action of UCPs, including UCP1 [5]–[8], some results do not confirm the involvement of UCPs as oxidative stress response proteins [9]. What is interesting, recently it has been proposed that UCP2 can function as a metabolite (malate, oxaloacetate, and aspartate) transporter [10]. The UCP2-mediated metabolite transport is coupled with the exchange for phosphate plus a proton. Thus, taking into account earlier reports focused on UCP2 function [4], [7], proton transport through the UCP2 could be coupled with the metabolite transport or not. Other carriers of IMM, e.g. ANT, also mediate metabolite transport and mitochondrial uncoupling [2]. In turn, ANT-dependent adenine nucleotide turnover (the exchange of intramitochondrial ATP for extramitochondrial ADP) is necessary to maintain OXPHOS in mitochondria. In addition to the basic function of ANT, this carrier can also mediate proton leak and behave like a classic uncoupling protein [2]. UCP-mediated proton leak and ANT-mediated proton leak need to be finely controlled to prevent ATP depletion in the cell. UCPs, as well as ANT, are stimulated by free fatty acids [1], [11] and hydroxynonenal, a membrane lipid peroxidation end product [12], though the inhibition of futile protein-mediated (ANT- or UCP-mediated) proton conductance is crucial for efficient ATP synthesis in mitochondria. Carboxyatractyloside (CATR), a specific inhibitor of ANT, inhibits purine nucleotide transport and ANT-mediated proton leak [11]; interestingly, ANT even fully inhibited by CATR can still contribute to the proton conductance [13]. Purine nucleotides (PNs) were first recognized as specific inhibitors of UCPs [1], and PN-dependent inhibition of respiratory rate accompanied by the restoration of ΔΨ is considered diagnostic of UCP function in isolated mitochondria. However, unlike the ANT-mediated proton conductance, which can be inhibited by ADP and GDP [11], [14], [15], UCPs are strongly inhibited by ATP and GTP, in addition to ADP and GDP. Therefore, because of this difference in specificity, the inhibition of mitochondrial proton conductance by PNs other than ADP and GDP should be considered diagnostic of UCP function [16]. ANT specializes in ADP and ATP transport across IMM, but nucleotide analogs may bind to the ANT without being transported [2]. For example, GDP is considered a weak binding competitor for ANT. Although GDP and GTP are known to accumulate in the mitochondrial matrix, the specific and ANT-independent mechanism of guanine nucleotide import into mitochondria is poorly described [17]–[19].

Interpretations of the effect of GDP on isolated mitochondria often omit at least two very important phenomena, namely: (i) the presence of nucleoside-diphosphate kinase (NDPK) in mitochondria, e.g., in the intermembrane space [20] and (ii) the possibility of GDP oxidative phosphorylation in mitochondria [21], [22]. NDPK is an enzyme that catalyzes the transfer of a γ-phosphate group from ATP (and other nucleoside triphosphates) to nucleoside diphosphates, e.g., ATP + GDP → ADP + GTP [23]. In mammals, only the NDPK-D isoform possesses a mitochondrial targeting sequence and is ubiquitously expressed, with the highest expression in liver, kidney, bladder, and prostate [20]. Thus, in light of the common usage of GDP in studies of UCP-mediated and ANT-mediated uncoupling, mitochondrial GDP turnover, i.e., GDP accumulation in the mitochondrial matrix [17]–[19], GDP transphosphorylation [23], or GDP oxidative phosphorylation [21], [22], complicate the interpretation of the GDP inhibitory effect on protein-mediated mitochondrial uncoupling.

Most functional studies on the activity of UCPs have been performed in non-phosphorylating (state 4) respiration, i.e., in the presence of oligomycin (an inhibitor of F_O_F_1_-ATP synthase), which prevents mitochondrial OXPHOS. However, these assays with isolated mitochondria do not reflect physiological conditions in which phosphorylating (state 3) respiration and non-phosphorylating (state 4) respiration are mixed. The main objective of the present work was to elucidate the action of GDP and GTP in isolated mammalian mitochondria under physiological-like conditions, i.e., those favoring OXPHOS (without the inhibitors CATR and oligomycin) and in the presence of ATP, which is abundantly synthesized in mitochondria. We suggest that under such conditions, the GDP-dependent inhibition of UCP should be severely weakened because of the action of NDPK and/or because of direct GDP import into the mitochondrial matrix, enabling its oxidative phosphorylation.

## Materials and Methods

### Animals

The experiments were carried out on adult 8-10-week-old male Wistar rats weighting 250–350 g. The animals were bred in the animal house at the Poznan University of Medical Sciences, Poznan, Poland. They were given free access to water and pellet food and were housed under standard humidity and temperature conditions on a 12 h light/dark cycle. Experimental protocols involving animals, their surgery and care were approved by the Local Ethics Committee on Animal Experimentation in Poznan, Poland (Permit Number: 15/2013) and were in compliance with the guidelines of the European Community Council Directive 2010/63/UE of 22 September 2010 on the protection of animals used for scientific purposes. Animals were sacrificed, and all efforts were made to minimize suffering. The Local Ethics Committee on Animal Experimentation in Poznan, Poland (Permit Number: 15/2013) approved this study.

### Isolation of mitochondria

For each experiment, kidney mitochondria were isolated from three rats. Isolation procedure was performed on ice in the cold room. The kidneys were washed, comminuted, and homogenized by four passes with a glass-Teflon homogenizer in ice-cold isolation medium containing 100 mM sucrose, 100 mM KCl, 50 mM Tris-HCl, 1 mM KH_2_PO_4_, 0.1 mM EGTA, 0.5 mM EDTA, and 0.2% fatty acid-free bovine serum albumin (BSA), pH 7.2. The presence of BSA in the isolation medium allowed the endogenous free fatty acids to be chelated from the homogenate suspension. The homogenate was filtered through sterile gauze, and the filtrate was centrifuged at 700 x g for 10 min at 4°C. The supernatant was centrifuged at 10 000 x g for 10 min at 4°C. Low-speed centrifugation at 700 x g for 10 min at 4°C preceded a final high-speed centrifugation at 8 000 x g for 10 min at 4°C. The final mitochondrial pellet was resuspended in ice-cold storage buffer containing 225 mM mannitol, 75 mM sucrose, 0.1 mM EDTA, and 10 mM Tris-HCl, pH 7.2. The mitochondrial protein concentration was determined by the biuret method with BSA as a standard.

Human endothelial cells (line EA.hy926, ATCC CRL-2922) were cultured, and their mitochondria were isolated as previously described [24].

### Preparation of inside-out submitochondrial particles (SMP) from rat kidney mitochondria

The inside-out SMP were prepared according to published procedures with some modifications [25], [26]. The pellet of rat kidney mitochondria was resuspended in ice-cold high-salt medium (225 mM mannitol, 75 mM sucrose, 10 mM Tris-HCl, and 20 mM MgCl_2_, pH 7.2.) to a final volume of approximately 4 ml with 10 mg protein/ml. Mitochondria sonication was done in a glass beaker (with a flat bottom) placed in an ice bath with a Bandelin Sonopuls sonifier for four 10 s bursts (50% of power) separated by a 1 min cooling period. After mitochondria disruption, the sample was diluted to approximately 20 ml with the medium used for sonication and centrifuged at 8 000 x g for 10 min at 4°C. The relatively intact mitochondria from the pellet were resuspended in a high-salt medium and further disrupted by a repetition of the sonication and pelleting procedure described above. The resulting two supernatants were then centrifuged at 20 000 x g for 10 min at 4°C to pellet intact mitochondria and large membrane fragments. Finally, supernatants were centrifuged at 105 000 g for 60 min at 4°C (Beckman Coulter Optima XPN-100 Ultracentrifuge, 70Ti rotor). The resulting pellets (SMP) were rinsed twice to reduce salt content, pooled and resuspended in ice-cold buffer containing 225 mM mannitol, 75 mM sucrose, and 10 mM Tris-HCl, pH 7.2. The SMP protein concentration was determined by the Bradford method with BSA as a standard.

### Measurement of mitochondrial respiration and membrane potential

Oxygen uptake was measured polarographically using a Clark-type oxygen electrode (Rank Brothers, Cambridge, UK) in 2.8 ml of incubation medium, with 2 mg of mitochondrial protein. The isolated rat kidney mitochondria were incubated at 37°C in a medium containing 10 mM KCl, 225 mM mannitol, 75 mM sucrose, 10 mM Tris-HCl, 5 mM KH_2_PO_4_, 0.18 mM MgCl_2_, 0.5 mM EDTA, and 0.1% BSA, pH 7.2. Isolated human endothelial cell mitochondria were incubated at 37°C in a medium containing 50 mM KCl, 70 mM sucrose, 10 mM Tris-HCl, 10 mM HEPES, 2.5 mM KH_2_PO_4_, 0.2 mM MgCl_2_, 1.5 mM EGTA, and 0.1% BSA. Succinate (5 mM) was used as an oxidizable substrate in the presence of rotenone (4 µM) to block electron input from complex I. As a control of mitochondrial quality, phosphorylating respiration was measured for each mitochondrial preparation to evaluate the coupling parameters. Only high-quality mitochondrial preparations, i.e., those with an ADP/O value of approximately 1.3 (with succinate as a respiratory substrate) and a respiratory control ratio of approximately 3.6–3.9, were used in the experiments. The values of O_2_ uptake are given in nanomoles of O per minute per milligram of protein. The mitochondrial membrane electrical potential (ΔΨ) was measured simultaneously with oxygen uptake using a tetraphenylphosphonium (TPP^+^)-specific electrode, as previously described [16]. The values of ΔΨ are given in millivolts (mV).

### Measurement of SMP respiration

Oxygen uptake was measured polarographically with a Clark-type oxygen electrode (Hansatech Instruments, UK) at 37°C in 0.6 ml of incubation medium (225 mM mannitol, 75 mM sucrose, 10 mM Tris-HCl, 5 mM KH_2_PO_4_, 0.18 mM MgCl_2_, 0.5 mM EDTA, and 0.1% BSA, pH 7.2) with 0.4 mg of SMP protein. The exogenous NADH oxidation (having no place in intact mammalian mitochondria), which was almost completely sensitive to rotenone, as well as the insensitivity of ADP-stimulated and GDP-stimulated respiration to carboxyatractyloside confirmed that our SMP were indeed inside-out SMP.

### Phosphorylating respiration measurements

**Figure 1:**
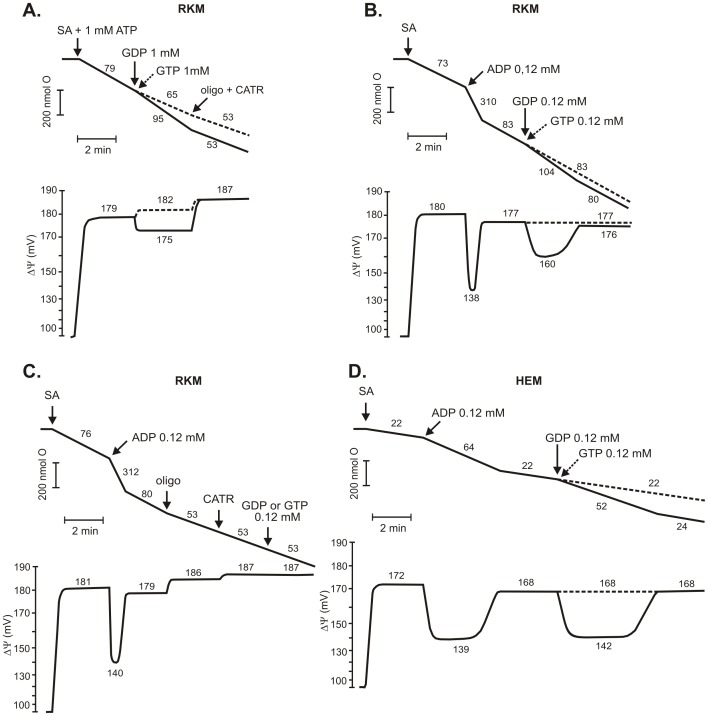
Effects of high (1 mM) and low (0.12 mM) concentrations of GDP on the respiratory rate and ΔΨ in mitochondria isolated from rat kidney (RKM) and human endothelial cells (HEM). The dashed line traces show the measurements obtained in the presence of GTP rather than GDP. Additions (where indicated): 5 mM succinate (SA), 0.12 mM or 1 mM nucleotides (GDP, GTP, or ADP), 0.7 µg/ml oligomycin, 3.6 µM CATR. (A) Effects of 1 mM GDP (or GTP) on rat kidney mitochondria in the presence of 1 mM ATP in the incubation medium. (B, C) Effects of 0.12 mM GDP (or GTP) on rat kidney mitochondria, which phosphorylated 0.12 mM ADP, in the absence (B) or presence of oligomycin and CATR (C). (D) Effects of 0.12 mM GDP (or GTP) on human endothelial mitochondria, which phosphorylated 0.12 mM ADP, in the absence of OXPHOS inhibitors. The numbers on the traces refer to the O_2_ consumption rates in nmol O/min/mg protein or to the ΔΨ values in mV. Representative measurements obtained at least from 5 different mitochondrial preparations are shown.

**Figure 2:**
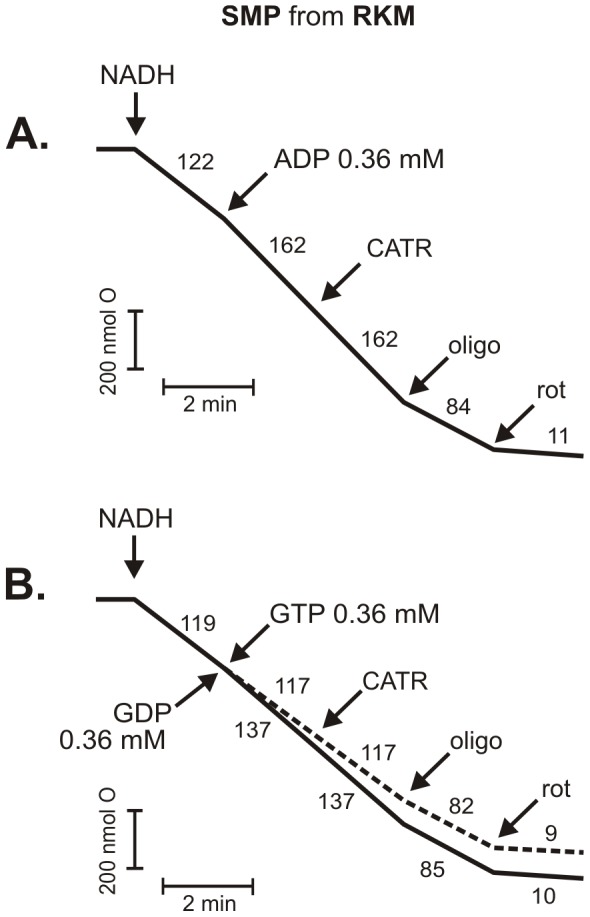
The effect of purine nucleotides on respiratory rate of SMP prepared from rat kidney mitochondria (RKM). (**A**) The stimulatory effect of 0.36 mM ADP. (**B**) The stimulatory effect of 0.36 mM GDP. No effect of GTP (dashed line). Additions (where indicated): 5 mM NADH, 3.6 µM CATR, 0.7 µg/ml oligomycin, and 4 µM rotenone (rot). The numbers on the traces refer to the O_2_ consumption rates in nmol O/min/mg protein. Representative measurements obtained at least from 5 different SMP preparations are shown.

The ADP/O ratio was determined by an ADP pulse method using 400 nmol of ADP for intact mitochondria. The total amount of oxygen consumed during phosphorylating respiration was used to calculate the ratio. The simultaneous measurements of ΔΨ enabled the fine control of the duration of state 3. It was necessary to incubate isolated mitochondria with ADP or ATP to observe the OXPHOS-like effect after GDP addition (Figs. 1A, 1B, 1D and Fig. S1A in File S1); in the case of ADP, GDP was added in the post-ADP state 4. The maximal GDP-induced OXPHOS-like effect in intact mitochondria required the equivalent concentration of ADP or ATP and was observed at a low GDP concentration, i.e., 120 µM (Fig. 1). On the contrary, the GDP-dependent stimulation of oxygen uptake in SMP did not require the presence of ATP (Fig. 2). However, the ADP-stimulated respiration and the GDP-stimulated respiration in SMP was much weaker compared to intact rat kidney mitochondria. It was not surprising, because it is generally agreed that SMP can be less coupled than intact mitochondria based on facts that usually in SMP: (i) ADP/O ratios are very low, (ii) respiratory control (state 3-state 4 transition) is barely observed and (iii) the ATPase activity is very high [27]. What is more, the same authors claim that the high rate of the backflow of protons *via* ATPase which is oligomycin-insensitive (“intrinsic uncoupled” activity of ATPase) significantly affects the apparent coupling in OXPHOS experiments with SMP.

### Proton leak measurements

**Figure 3:**
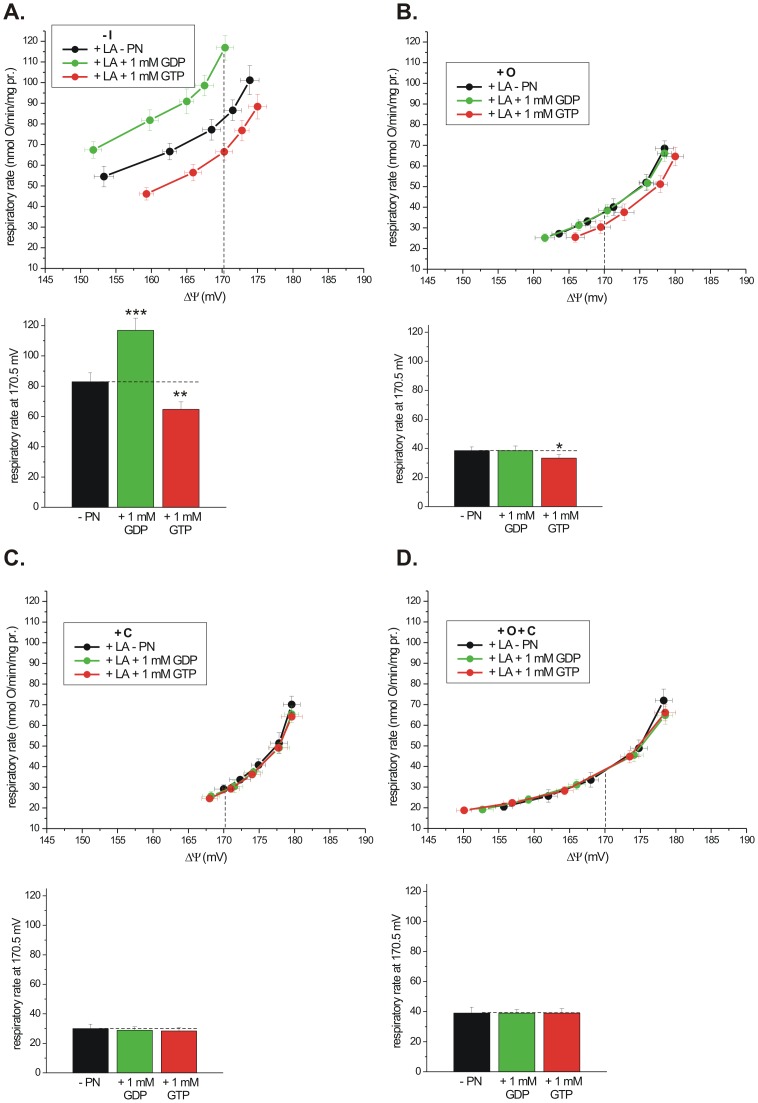
The effect of GDP (1 mM) and GTP (1 mM) on proton leak of isolated rat kidney mitochondria under phosphorylating and non-phosphorylating conditions in the presence of linoleic acid (LA, 25 µM). The relationships between the respiratory rate and ΔΨ (proton leak kinetics) are shown. The measurements were performed in the absence of OXPHOS inhibitors (−I) (A), in the presence of oligomycin (+ O) alone (B), in the presence of CATR (+ C) alone (C), and with the simultaneous presence of both inhibitors (+ O + C) (D). The oxidation of succinate (5 mM) was gradually decreased by increasing the concentration of malonate (0.5–1.4 mM). The mitochondria (2 mg) were incubated in 2.8 ml of incubation medium supplemented with rotenone (4 µM) and ATP (0.8 mM). The inserts of column plots show the respiratory rates at the highest common ΔΨ (170.5 mV) for the same dataset. The values are the means ± S.D. of 6 independent experiments (mitochondrial isolations).

The response of proton conductance to its driving force can be expressed as the relationship between the oxygen consumption rate and ΔΨ (a flux-force relationship) when varying the membrane potential by titration with respiratory chain inhibitors. The proton leak kinetics (Fig. 3 and Fig. S1 in File S1) were examined in the presence of 0.8–1 mM ATP under four different conditions: (i) in the absence of CATR and oligomycin, (ii) in the presence of CATR (3.6 µM) alone, (iii) in the presence of oligomycin (0.7 µg/ml) alone, and (iv) with the simultaneous presence of CATR and oligomycin. To induce UCP activity, linoleic aid (LA) was used at a concentration of 25 µM (with 2 mg of mitochondrial protein in 2.8 ml of incubation medium). LA was always added on stabilized state 4 respiration (in the absence or presence of OXPHOS inhibitors). GDP and GTP were added to 1 mM, and always after LA. The respiratory rate and ΔΨ were varied by modulating the coenzyme Q-reducing pathway with malonate (0.3–1.6 mM), a competitive inhibitor of succinate dehydrogenase. To assess the statistical significance of the induced shifts in the proton leak curves, we generally compared the respiration rates at the highest common ΔΨ values for pairs of curves from 5–7 independent experiments using Student's *t*-test for unpaired data.

### Statistical analysis

The results are presented as the means ± S.D. obtained from at least 5 mitochondrial isolations, with each determination performed at least in duplicate. An unpaired two-tailed Student's *t*-test was used to identify significant differences; in particular, differences were considered to be statistically significant if *p*<0.05 (*), *p*<0.01 (**), or *p*<0.001 (***).

### Chemicals

Purine nucleotides (GTP, GDP, ATP, and ADP), LA, oligomycin, and CATR were obtained from Sigma-Aldrich (St. Louis, MO, USA). LA and oligomycin were dissolved in methanol. The LA stocks were stored at −80°C under nitrogen and prepared in brown glass vials.

## Results and Discussion

### A high concentration of GDP (1 mM) stimulates the respiratory rate and decreases the membrane potential of isolated mitochondria

The addition of GDP and other nucleoside diphosphates can stimulate respiration in isolated mitochondria when measurements are performed in the absence of OXPHOS inhibitors (oligomycin and/or CATR) [23], [28]. This phenomenon can be explained as a result of NDPK activity, which can be bound to IMM [29]. Thus, in the presence of GDP and ATP, there is a possibility for local ADP regeneration as a consequence of the transphosphorylation reaction catalyzed by NDPK and the subsequent stimulation of OXPHOS [23]. Using mitochondria isolated from rat kidneys and human endothelial cells, we also observed the stimulation of the respiratory rate accompanied by a decrease in ΔΨ in the absence of OXPHOS inhibitors, but this effect occurred with a much higher GDP concentration, than previously described. The phenomenon can be observed in traces of simultaneous measurements of respiratory rate and ΔΨ (Figs. 1A, 1B and 1D) and flux-force relationships, (Fig. 3A and Fig. S1A in File S1). Specifically, in the presence of 1 mM ATP, even a concentration of 1 mM GDP could induce a state 4-state 3 transition that was sensitive to CATR and oligomycin (Fig. 1A). However, as with 1 mM ADP, the state 3-state 4 transition, revealing the cessation of OXPHOS, was not observed with 1 mM GDP (data not shown).

Previous studies using isolated mammalian mitochondria have shown that, in contrast to other nucleoside diphosphates, GDP acts both as a substrate (up to 0.15 mM) and an inhibitor (above 0.15 mM) of NDPK, with almost complete inhibition by 0.6 mM GDP [23], [30], [31]. In our experiments, using 120 µM GDP (not inhibitory for NDPK), the stimulation of respiration (state 4-state 3 transition), followed by the return to basal respiration (state 3-state 4 transition), was clearly observed in the human endothelial mitochondria and rat kidney mitochondria (Figs. 1B and 1D). As shown for the rat kidney mitochondria, 1 mM GDP was still stimulating, though its effect revealed a weaker acceleration of the respiratory rate accompanied by a much smaller decrease in ΔΨ (Fig. 1A) in comparison to the conditions with a lower (120 µM) GDP concentration (Fig. 1B). Similar results were obtained using the human endothelial cells mitochondria (data not shown). In contrast to GDP, 1 mM GTP inhibited the respiratory rate and increased ΔΨ in the absence of OXPHOS inhibitors, revealing an inhibition of mitochondrial uncoupling most likely mediated by UCP (Fig. 1A, dashed traces). It also means that in tested mitochondria, GTP is poorly susceptible to dephosphorylation and is not a substrate for NDPK.

Because the action of mitochondrial NDPK is most likely fully inhibited in the presence of 0.6 mM GDP [23], [30], the observed 1 mM GDP-induced state 4-state 3 transition (Figs. 1A, 3A and Fig. S1 in File S1) could be a result of GDP import into the mitochondria [17]–[19], followed by the GDP oxidative phosphorylation [21], [22] rather than NDPK-dependent GDP transphosphorylation. However, previous studies on ANT specificity toward PN exchange have excluded guanine nucleotide transport [17], [32], [33]. Moreover, it has been shown that the transport of ADP through ANT can be competitively inhibited by GDP [34]. Nevertheless, GDP is readily taken up and concentrated in the matrix of isolated mitochondria through at least two distinct mechanisms, i.e., atractyloside-sensitive and -insensitive pathways [17], [19].

Similar to a previous report [14], in the absence of an ATP or ADP prepulse, we did not observe the state 4-state 3 transition upon the addition of GDP (data not shown). The matrix ATP pool, generated upon ADP prepulse or direct ATP addition, was crucial for the GDP-induced OXPHOS-like effect in the absence of oligomycin and CATR (Figs. 1A, 1B, 1D, 3A and Fig. S1A in File S1). Similar to ADP, extramitochondrial ATP is an effective ligand for the exchange of intramitochondrial ATP *via* ANT. The uptake of ATP can also be catalyzed by the mitochondrial ATP-Mg/P_i_ transporter that is little sensitive to CATR [35]. Thus, extramitochondrial ATP can effectively generate the matrix pool of ATP, which in turn might be exchanged with exogenous GDP *via* carrier-mediated transport. Moreover, the maximal GDP-induced OXPHOS-like effect for a given GDP concentration was strictly dependent on the ATP (or ADP pulse) concentration and required a 1∶1 ratio (Figs. 1A, 1B, and 1D). This observation supports the idea that the putative carrier-mediated uptake of GDP could be coupled to the efflux of a counter-substrate and resembles ADP transport through ANT in exchange for ATP. Additionally, it must be kept in mind that OXPHOS of GDP is most likely conserved in all organisms. Bacterial F_O_F_1_-ATPase [36] and mammalian mitochondrial F_O_F_1_-ATP synthase [21], [22] have an affinity to bind GDP and phosphorylate it to produce GTP.

A smaller decrease in ΔΨ and a weaker increase in the respiratory rate caused by GDP compared to ADP (Figs. 1B and 1D) could reflect the much lower affinity of ANT as well as F_O_F_1_-ATP synthase for GDP compared to ADP [2], [21], [22]. The failure to observe GDP-induced stimulation of the respiratory rate accompanied by a decrease in ΔΨ in the presence of CATR (Figs. 1C, 3C and Fig. S1C in File S1) might indicate the involvement of ANT in GDP translocation or, alternatively, an as-yet-unknown CATR-sensitive carrier. Irrespectively of the GDP concentration, the GDP effect was also sensitive to oligomycin (Figs. 1A, 1C, 3B and Fig. S1B in File S1) and required the presence of inorganic phosphate (data not shown), indicating the induction of OXPHOS upon GDP addition. It must be stressed that, under our experimental conditions, none of the applied GTP concentrations could induce the state 4-state 3 transition that is characteristic of the GDP OXPHOS-like effect in the absence of OXPHOS inhibitors (Figs. 1, 3A and Fig. S1A in File S1).

Ideal conditions to test the potency of GDP to induce OXPHOS without the risk of its transphosphorylation should exclude the action of NDPK. Unfortunately, we could not switch off the activity of NDPK to determine the effect of GDP addition because one of the known inhibitors of NDPK, 3′-azido-3′-deoxythymidine (AZT) [30], is also a strong inhibitor of ANT [37]. In turn, cromoglycate, also identified as an NDPK inhibitor, in as high a concentration as 10 mM can block NDPK catalytic activity by only 60% [38]. Therefore, the only way to completely preclude NDPK-dependent transphosphorylation without switching off ANT is to use a higher concentration of GDP [23], [30]. Additionally, the usage of labeled GDP will not clarify where the newly synthesized GTP arose: GTP as a product of F_O_F_1_-ATP synthase activity resides in the mitochondrial matrix, where NDPK is also located [29]. The same could be true for GTP arising through NDPK activity bound to IMM but facing the mitochondrial intermembrane space, as GTP from this site can be transported to the matrix [19]. Thus, by performing measurements under conditions favoring OXPHOS, it is difficult to unambiguously determine whether the stimulation of respiration in isolated mitochondria upon GDP addition in the presence of ATP, particularly at lower GDP concentrations when NDPK is active, is merely the result of NDPK activity or due to GDP import into the mitochondria. The GDP-dependent stimulation of respiration (the state 4-state 3 transition) is most likely a result of a mixture of these two phenomena, a mixture of ADP (a product of NDPK activity) and GDP oxidative phosphorylation. Experiments with inside-out SMP (from rat kidney mitochondria) supported GDP OXPHOS phenomenon (Fig. 2). In SMP, ADP and GDP stimulated the respiratory rate, while GTP was ineffective. However, the potency of both nucleotides to stimulate respiration decreased significantly in SMP compared to intact mitochondria. Nonetheless, the ADP effect was twice as strong as GDP effect. The GDP effect was observed in the absence of ATP, excluding the involvement of NDPK activity. The addition of CATR did not inhibit the ADP- and GDP-induced acceleration of respiratory rate. Thus, the ADP and GDP effects were not governed by transporter systems (e.g., ANT). However, these effects were abolished by oligomycin, indicating the involvement of F_O_F_1_-ATP synthase. Although experiments with SMP indicated GDP OXPHOS in mitochondria, SMP model did not clarify the involvement of GDP/GTP transport in the observed phenomenon.

### Different effects of GDP and GTP on LA-induced mitochondrial proton leak in the absence of OXPHOS inhibitors

UCP and ANT are considered as two main catalysts of futile proton leak in mammalian mitochondria [12]–[14]. The presence of UCP2 in isolated rat kidney and human endothelial cell mitochondria was confirmed by western blot analysis (Fig. S2 in File S1). However, the effect of GDP and GTP on LA-induced mitochondrial respiration under conditions favoring OXPHOS is poorly recognized. Employing four different incubation conditions, i.e., in the presence of ATP (i) with no OXPHOS inhibitors, (ii) with oligomycin alone, (iii) with CATR alone, and (iv) with both inhibitors together, we studied different effects of GDP and GTP on rat kidney mitochondrial proton leak by analyzing flux-force relationships in the presence of LA, an activator of UCP (Fig. 3). The LA effect was comparable in the absence or presence of OXPHOS inhibitors. We used a 1 mM concentration of guanine nucleotides to provide optimal conditions for UCP inhibition and to attenuate the process of transphosphorylation catalyzed by NDPK [23], [30]. Under conditions favoring OXPHOS, in the absence of oligomycin and CATR, the effects of GDP and GTP on LA-induced proton leak differed diametrically (Fig. 3A). Namely, the effect of GDP was stimulatory, and the effect of GTP was inhibitory. Similar results were obtained in the absence of LA, i.e., for non-induced proton leak (Fig. S1A in File S1). Column plots (Fig. 3) present the values of the respiratory rates reflecting proton leak at the highest common ΔΨ, i.e., at 170.5 mV for the four studied conditions. In the presence of 25 µM LA, 1 mM GDP increased the respiratory rate by approximately 41%, and 1 mM GTP decreased the respiratory rate by approximately 22% (Fig. 3A). The stimulating effect of GDP, under conditions favoring OXPHOS, was observed in the absence or presence of linoleic acid (Figs. 1, 3A and Fig. S1A in File S1), thus OXPHOS system and NDPK activity seem to be unaffected, at least by used LA concentration (25 µM). The LA-induced proton leak, in the absence of OXPHOS inhibitors, was fully inhibited by GTP when the Q-reducing pathway was gradually decreased with malonate (Fig. 3A), as previously described [16], [39], [40], what is diagnostic for UCP action. Thus, it may be concluded that among potential carriers of IMM involved in uncoupling process [2], UCP is the main catalyst of LA-induced proton leak, at least in rat kidney mitochondria under physiological-like conditions.

In turn, under conditions preventing OXPHOS, the stimulatory effect of GDP on LA-induced proton leak was completely abolished and the inhibitory effect of GTP was seriously weakened (Figs. 3B, 3C, and 3D). Similar results were obtained in the absence of LA, i.e., for non-induced proton leak (Figs. S1B, S1C, and S1D in File S1). The attenuation of PNs inhibitory effect in the presence of OXPHOS inhibitors could mean that both inhibitors, oligomycin and CATR, most likely exhibit a nonspecific action on UCP and impair the PN-dependent inhibition of UCP but not UCP-dependent proton leak. These data support many results showing moderate or no GDP-dependent inhibitory effect of proton conductance in the presence of OXPHOS inhibitors [12], [14], [15], [41]–[45]. It is likely that CATR induces a conformational change in UCP, which partly or fully prevents the PN-dependent inhibition of UCP. An analogous explanation has been proposed for ANT [46]. Namely, the structure change of ANT under the influence of hydroxynonenal likely desensitizes the ANT-mediated proton leak to CATR. However, because in our conditions, LA was still stimulatory in the presence of OXPHOS inhibitors and this LA-induced uncoupling was poorly sensitive to guanine nucleotides, a LA-stimulated component (likely carrier), which is insensitive to GDP, GTP and CATR, could mediate the proton conductance, as previously proposed for hydroxynonenal-induced uncoupling [45]. On the contrary, the LA-induced GTP/GDP-insensitive uncoupling in the presence of CATR could involve ANT, because ANT even fully inhibited by CATR can still contribute to the proton conductance [13]. If a CATR-insensitive domain of ANT stimulates proton leak in the presence of LA, and this domain is insensitive to guanine nucleotides (as observed in this study, Fig. 3C), it indicates that guanine nucleotides do not function as inhibitors of ANT-mediated uncoupling, as previously described [11], [45]. On the other hand, efficient guanine nucleotide-dependent inhibition of proton leak in the presence of CATR and oligomycin has been found for many UCP homologues [12], [16], [24], [40], [41], [47], [48]. Alternatively, the suppression of PNs inhibitory effect, especially in the presence of oligomycin alone, could be explained as a result of guanine nucleotides binding to ANT, proposed for GDP [8], [14], [15], [34], and/or their import into the mitochondrial matrix, proposed for GDP and GTP [17]–[19], what certainly lowers the PNs concentration in the mitochondrial intermembrane space where UCP faces its PN-binding site [1].

The specificity of GTP and GDP action in the absence or presence of LA under conditions favoring OXPHOS, as described in detail above for isolated rat kidney mitochondria, is also characteristic in mitochondria isolated from human endothelial cells (data not shown).

### General discussion

The incubation of isolated mitochondria under non-phosphorylating conditions (in the presence of oligomycin and/or CATR) limits the interpretation of the physiological regulation of UCP isoforms and ANT, two major catalysts of the proton conductance in mitochondria [12]–[14]. In recent years, the non-additivity of GDP (the most widely used PN for UCP inhibition) and CATR (thus far considered to be highly specific only for ANT inhibition) has been reported for the inhibition of mitochondrial proton leak [12], [15], [45]. Moreover, the potential promiscuity of GDP and CATR influences the inhibitory effect of ANT-mediated and UCP-mediated proton leak [12], [15], [44], [45], [49]. Therefore, the indirect (or nonspecific) interaction of CATR with UCP was proposed [12], [15], [45]. Once UCP is inhibited by CATR, any additional inhibition by PN would not occur. A strongly limited guanine nucleotides effect in the presence of CATR (Fig. 3C and Fig. S1C in File S1) could be explained as follows: (i) CATR, irrespectively of the fatty acid presence, nonspecifically binds with UCP and impairs the PN-dependent inhibition of UCP (desensitization of UCP to nucleotides by CATR) but not UCP-dependent proton leak, (ii) IMM carriers other than UCP and ANT are stimulated by fatty acids and catalyze proton leak, which is insensitive to GDP, GTP and CATR; in this case, CATR could be considered not only as inhibitor of ANT-mediated uncoupling but also UCP-mediated uncoupling, (iii) fatty acid-induced GTP/GDP-insensitive uncoupling in the presence of CATR is catalyzed both by CATR-insensitive ANT domain [13] and desensitized to nucleotides (by CATR) UCP, and finally, (iv) carrier-independent uncoupling mechanisms mediate the proton leak. If CATR inhibits UCP, the effect is rather nonspecific (or indirect), because bongkrekate (another specific inhibitor of ANT) also likely inhibits the UCP-dependent proton conductance [15]. The inhibitory effect of CATR towards other carrier (citrate carrier) than ANT has also been reported [50]. However, it has also been reported that CATR does not influence the GDP binding to UCP2 [12] and does not inhibit UCP1 action [34]. In summary, if CATR and GDP are not specific for their classical targets, measurements focused on UCP inhibition should not be performed in the presence of GDP and CATR.

By creating *in vitro* physiological-like conditions, i.e., excluding CATR and oligomycin from the incubation medium supplemented with ATP, we identified another reason not to use GDP for UCP inhibition. For the first time at the level of mitochondrial functional studies using mitochondria isolated from rat kidney and human endothelial cells, we demonstrate that a high concentration of GDP (1 mM) stimulated the respiratory rate and decreased ΔΨ in the presence of LA, a potent activator of UCP isoforms (Fig. 3A). We observed exactly the opposite effect than what was expected for UCP inhibition, whereby the recoupling effect was revealed as a respiratory rate decrease accompanied by the restoration of ΔΨ. The effect of 1 mM GDP was more similar to ADP OXPHOS in which oxygen consumption is increased and ΔΨ decreases (Figs. 1A, 1B and 1C). This is a rather new perspective of the action of GDP in mitochondria, taking into account its common usage as an inhibitor of UCP-mediated proton leak. The state 4-state 3 transition with 1 mM GDP, in the absence of OXPHOS inhibitors, was also induced in the absence of LA (Fig. S1 in File S1). Moreover, under conditions favoring OXPHOS and in the presence of ATP, another guanine nucleotide commonly used for UCP inhibition, i.e. GTP, produced the expected effects diagnostic for UCP inhibition, regardless of the presence of LA (Figs. 1A, 3A and Fig. S1A in File S1).

**Figure 4:**
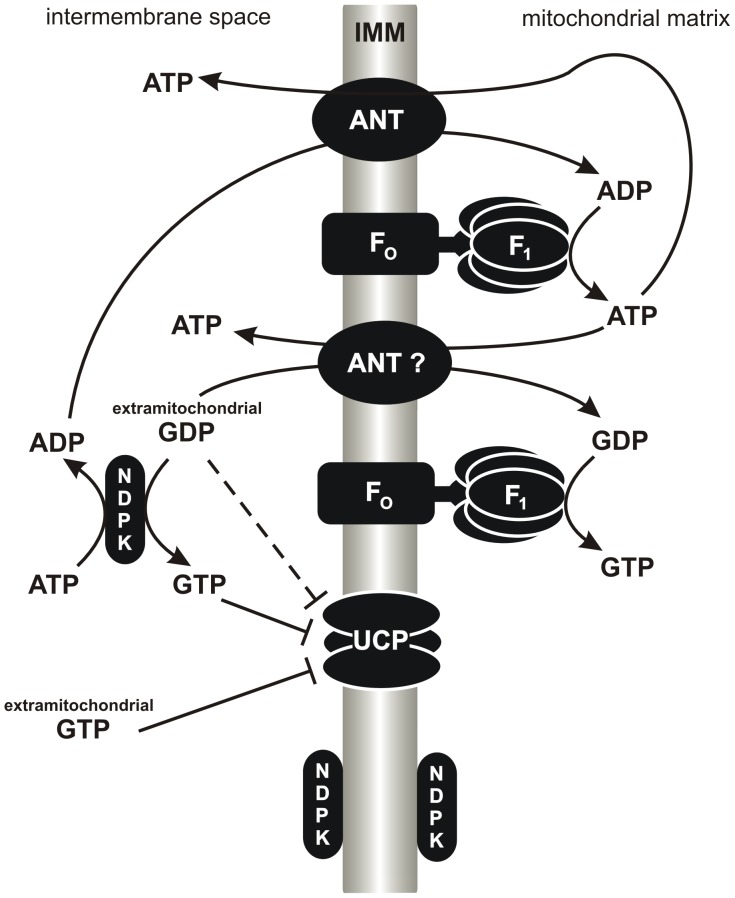
Model of GDP and GTP action in mitochondria. Extramitochondrial GDP can be transphosphorylated to GTP in the presence of ATP in a reaction catalyzed by mitochondrial NDPK. NDPK is bound to the cytosolic-facing or matrix-facing IMM leaflet but is shown separately for clarity. Alternatively, extramitochondrial GDP can be imported *via* ANT or another, as-yet-unknown, CATR-sensitive carrier into the mitochondrial matrix to enable its OXPHOS. In turn, extramitochondrial GTP, or GTP synthesized in the NDPK-dependent reaction, functions as the stronger UCP inhibitor (blunt-end solid arrow) than GDP (blunt-end dashed arrow).

The GDP-induced OXPHOS-like effect could be explained by NDPK-dependent transphosphorylation between GDP and ATP, which generates the ADP pool and subsequently induces OXPHOS (Fig. 4) [23], [28], [30]. However, another explanation might be needed, as NDPK was found to be sensitive to increasing concentrations of GDP, being almost completely inhibited at 0.6 mM GDP [23], [30], and taking into account the stimulatory effect of 1 mM GDP (Figs. 1A, 3A and Fig. S1A in File S1). The alternative explanation, in accordance with earlier studies [17], [19], [21], [22], [36], involves the possibility of the direct transport of GDP across IMM and its OXPHOS in the mitochondrial matrix as the 1 mM GDP stimulatory effect was completely abolished in the presence of OXPHOS inhibitors (Figs. 1, 3, 4 and Fig. S1 in File S1). CATR sensitivity of the GDP-induced OXPHOS-like effect (Figs. 1C, 3C and Fig. S1C in File S1) indicates that putative carrier-dependent GDP transport across IMM might be consistent with ANT or another, as-yet-unknown, CATR-sensitive guanine nucleotide carrier. This idea is supported by the atractyloside-sensitive pathway of guanine nucleotide transport in mammalian mitochondria [19]. However, the ANT involvement in GDP import is controversial [17], [32], [51]. On the other hand, it must be mentioned that, although GDP is considered to be a binding but not transported ligand for ANT, the clear differentiation of binding from a slow uptake has rarely been tested [2].

Generally, little is known about how GDP enters the mitochondrial matrix. In the yeast *Saccharomyces cerevisiae*, a specific GTP/GDP carrier has been described, however poorly affected by the powerful inhibitors of ANT, CATR and bongkrekate [52]. It has also been reported that the human mitochondrial deoxynucleotide carrier, which is partly sensitive to CATR, could be involved in GDP transport [53].

The purpose of enhanced GTP synthesis in the mitochondrial matrix, *via*, e.g., the GDP OXPHOS, could be due to GTP-dependent mitochondrial protein synthesis and the incorporation of GTP into various mitochondrial RNAs [54]. An important role of GTP in mitochondrial iron metabolism has also been considered [55]. GTP cannot arise through the conversion of inosine or adenine compounds or *via* uptake and synthesis from the salvage pathways using guanine or guanosine [18]. Thus, an additional source of GTP arising from GDP OXPHOS could complement the well-known pathway of GTP synthesis during the citric acid cycle and support the expression of the mitochondrial genome during intensive mitochondrial biogenesis in rapidly growing tissues. In the mitochondrial matrix, GTP may also arise through NDPK action [29], though this pathway consumes valuable ATP. In turn, GDP OXPHOS to GTP consumes inorganic phosphate, thereby favoring a high energy potential in the cell. Different pathways of GTP synthesis, such as the citric acid cycle, NDPK action, and GDP OXPHOS, could also play an important role during the fusion and fission of mitochondria and thus regulate GTP-dependent mitochondrial membrane dynamics [56]. The high concentration of GTP in mitochondria could also prevent UCP action and promote ATP synthesis *via* OXPHOS during periods of high ATP demand.

On the basis of our present study, the following main conclusions can be drawn. UCP, an IMM carrier specializing in futile proton conductance, and ANT or other CATR-sensitive PN carriers that mimic UCP action are differently regulated by PNs. A CATR-sensitive PN carrier (ANT or an as-yet-unknown carrier) and UCP could compete for GDP in the intermembrane space of mitochondria. However, in the case of UCP, the binding of GDP could lead to the inhibition of UCP action, whereas the CATR-sensitive PN carrier could mediate the transport of GDP, its substrate, across IMM. It must be considered that ANT and most likely other putative PN carriers require a 1∶1 exchange [2]. During many studies of proton leak kinetics, only one exogenous PN was typically used [8], [14], [15], [34], which could be the reason of the putative ANT inhibition by GDP. If ANT is involved in GDP import, GDP inhibition of ANT-sustained proton leak might be an artifact resulting from measurements performed in the presence of oligomycin; F_O_F_1_-ATP synthase arrested by oligomycin could cause GDP accumulation in the mitochondrial matrix and thus limit the carrier-mediated GDP import from the intermembrane space. Moreover, when ANT permanently mediated adenine nucleotide transport, no or a very weak competitive effect of GDP toward the transported ADP was revealed [14], [17], [28], [57]. Thus, it is difficult to consider GDP as a potent inhibitor of ANT, although the GDP inhibitory effect of ANT-mediated proton leak was observed [8], [14], [15], [34]. However, it must be mentioned that GDP was also reported to be a completely ineffective inhibitor of ANT-mediated uncoupling [11], [12], [45], [48], [58], [59]. The same might be true for ADP, which has also been described as an inhibitor of ANT-mediated proton leak [11], [48], [58], because the inhibitory effect of ADP was revealed in the presence of oligomycin, with ADP being transported *via* ANT but without being phosphorylated in the matrix (no activity of F_O_F_1_-ATP synthase). Besides GDP and ADP, some inhibition of free fatty acid-induced ANT-mediated uncoupling was found with long chain acyl-CoA [11]. However this kind of inhibition could have minor physiological significance [50]. Similar to other authors [11]–[13], [58], in the presence of oligomycin, we also observed an inhibition of the respiratory rate accompanied by ΔΨ restoration upon CATR addition (data not shown), indicating the contribution of ANT to mitochondrial protein-mediated proton leak. However, CATR, a glycoside synthesized by some plants (e.g., *Xanthium* species) [60], is not a physiological regulator of animal ANT. In summary, the highly specific physiological inhibitor of ANT-mediated proton leak or proton leak sustained through another CATR-sensitive carrier(s) is still not identified

Finally, our results suggest that, under physiological conditions, GTP could play the role of the strongest UCP inhibitor, stronger than GDP and ATP. Thus, GTP rather than GDP should be used in bioenergetics studies *in vitro* as diagnostic inhibitor of UCP function.

## Supporting Information

File S1 Figure S1, The effect of GDP (1 mM) and GTP (1 mM) on proton leak of rat kidney mitochondria in the absence of linoleic acid. The relationships between the respiratory rate and ΔΨ (proton leak kinetics) are shown. The measurements were performed in the absence of OXPHOS inhibitors (−I) (**A**), in the presence of oligomycin (+O) alone (**B**), in the presence of CATR (+C) alone (**C**), and with the simultaneous presence of both inhibitors (+O +C) (**D**). The oxidation of succinate (5 mM) was gradually decreased by increasing the concentration of malonate (0.3–1.6 mM). The mitochondria (2 mg) were incubated in 2.8 ml of incubation medium supplemented with rotenone (4 µM) and ATP (0.8 mM). The inserts of column plots show the respiratory rates at the highest common ΔΨ (175 mV) for the same dataset. The values are the means ± S.D. of 6 independent experiments (mitochondrial isolations). Figure S2, Western blot analysis of mitochondria isolated from rat kidney (RKM) and human endothelial cells (HEM) using anti-UCP2 (sc-6525, Santa Cruz Biotechnology) antibodies. Detection of mitochondrial marker (cytochrome oxidase, COXII, MS404, MitoScience) was performed as a control. Experimental conditions as in [24]. Different amounts of protein (50 or 100 µg) were loaded into each lane (as indicated).
(PDF)
