# Supplementary material for: Different Effects of Guanine Nucleotides (GDP and GTP) on Protein-Mediated Mitochondrial Proton Leak
Source: PLoS One. 2014 Jun 6;9(6):e98969. doi: 10.1371/journal.pone.0098969 (PMC4056835; doi:10.1371/journal.pone.0098969)
Supplement: File S1 — Figure S1, The effect of GDP (1 mM) and GTP (1 mM) on proton leak of rat kidney mitochondria in the absence of linoleic acid. The relationships between the respiratory rate and ΔΨ (proton leak kinetics) are shown. The measurements were performed in the absence of OXPHOS inhibitors (−I) (A), in the presence of oligomycin (+O) alone (B), in the presence of CATR (+C) alone (C), and with the simultaneous presence of both inhibitors (+O +C) (D). The oxidation of succinate (5 mM) was gradually decreased by increasing the concentration of malonate (0.3–1.6 mM). The mitochondria (2 mg) were incubated in 2.8 ml of incubation medium supplemented with rotenone (4 µM) and ATP (0.8 mM). The inserts of column plots show the respiratory rates at the highest common ΔΨ (175 mV) for the same dataset. The values are the means ± S.D. of 6 independent experiments (mitochondrial isolations). Figure S2, Western blot analysis of mitochondria isolated from rat kidney (RKM) and human endothelial cells (HEM) using anti-UCP2 (sc-6525, Santa Cruz Biotechnology) antibodies. Detection of mitochondrial marker (cytochrome oxidase, COXII, MS404, MitoScience) was performed as a control. Experimental conditions as in [24]. Different amounts of protein (50 or 100 µg) were loaded into each lane (as indicated). (PDF) [file pone.0098969.s001.pdf]

Figure S1

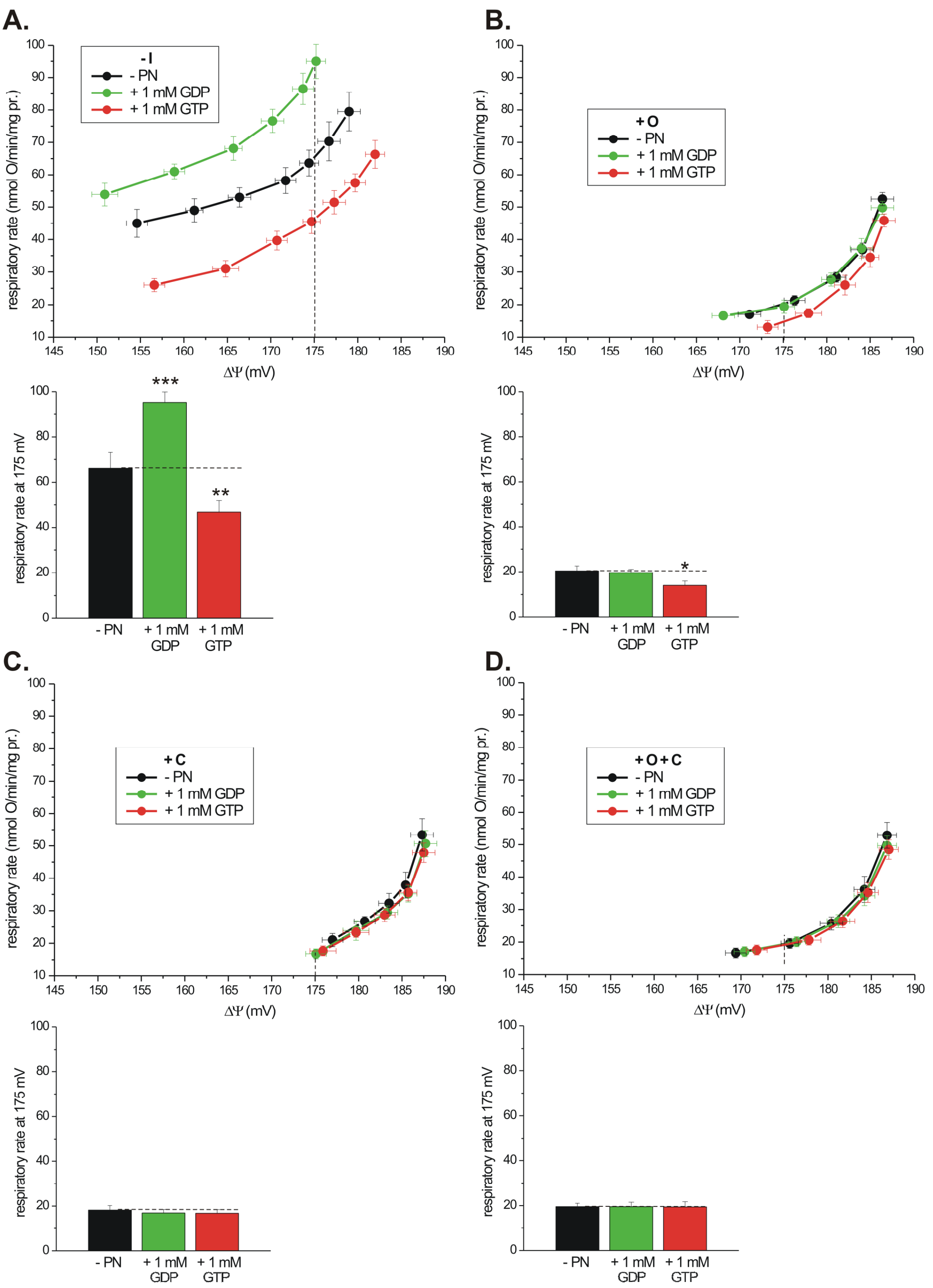

Figure S1. The effect of GDP (1 mM) and GTP (1 mM) on proton leak of rat kidney mitochondria in the absence of linoleic acid. The relationships between the respiratory rate and ΔΨ (proton leak kinetics) are shown. The measurements were performed in the absence of OXPHOS inhibitors (– I) (**A**), in the presence of oligomycin (+ O) alone (**B**), in the presence of CATR (+ C) alone (**C**), and with the simultaneous presence of both inhibitors (+ O + C) (**D**). The oxidation of succinate (5 mM) was gradually decreased by increasing the concentration of malonate (0.3-1.6 mM). The mitochondria (2 mg) were incubated in 2.8 ml of incubation medium supplemented with rotenone (4 µM) and ATP (0.8 mM). The inserts of column plots show the respiratory rates at the highest common ΔΨ (175 mV) for the same dataset. The values are the means ± S.D. of 6 independent experiments (mitochondrial isolations).

Figure S2

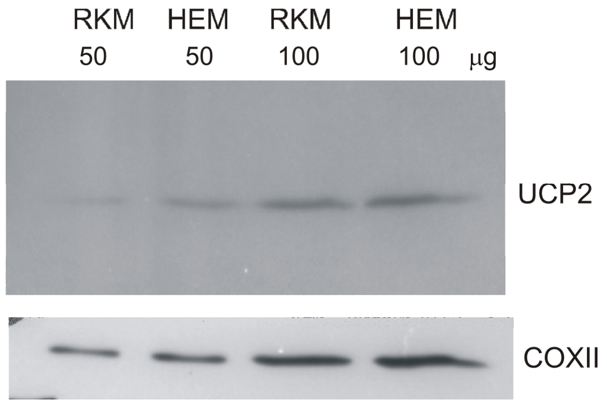

Figure S2. Western blot analysis of mitochondria isolated from rat kidney (RKM) and human endothelial cells (HEM) using anti-UCP2 (sc-6525, Santa Cruz Biotechnology) antibodies. Detection of mitochondrial marker (cytochrome oxidase, COXII, MS404, MitoScience) was performed as a control. Experimental conditions as in [24]. Different amounts of protein (50 or 100 μg) were loaded into each lane (as indicated).
